# Inferring protein from transcript abundances using convolutional neural networks

**DOI:** 10.1186/s13040-025-00434-z

**Published:** 2025-02-27

**Authors:** Patrick Maximilian Schwehn, Pascal Falter-Braun

**Affiliations:** 1 Institute of Network Biology (INET), Molecular Targets and Therapies Center (MTTC), Helmholtz Munich, Neuherberg, Germany; 2 Microbe-Host Interactions, Ludwig-Maximilians-Universität München, Planegg-Martinsried, Germany

**Keywords:** Translational regulation, Protein-to-mRNA ratio, Convolutional neural networks, Regression analysis, Explainable AI

## Abstract

**Background:**

Although transcript abundance is often used as a proxy for protein abundance, it is an unreliable predictor. As proteins execute biological functions and their expression levels influence phenotypic outcomes, we developed a convolutional neural network (CNN) to predict protein abundances from mRNA abundances, protein sequence, and mRNA sequence in *Homo sapiens (H. sapiens)* and the reference plant *Arabidopsis thaliana (A. thaliana)*.

**Results:**

After hyperparameter optimization and initial data exploration, we implemented distinct training modules for value-based and sequence-based data. By analyzing the learned weights, we revealed common and organism-specific sequence features that influence protein-to-mRNA ratios (PTRs), including known and putative sequence motifs. Adding condition-specific protein interaction information identified genes correlated with many PTRs but did not improve predictions, likely due to insufficient data. The integrated model predicted protein abundance on unseen genes with a coefficient of determination (r^2^) of 0.30 in *H. sapiens* and 0.32 in *A. thaliana.*

**Conclusions:**

For *H. sapiens,* our model improves prediction performance by nearly 50% compared to previous sequence-based approaches, and for *A. thaliana* it represents the first model of its kind. The model’s learned motifs recapitulate known regulatory elements, supporting its utility in systems-level and hypothesis-driven research approaches related to protein regulation.

**Supplementary Information:**

The online version contains supplementary material available at 10.1186/s13040-025-00434-z.

## Background

For the predictive analysis of biological systems and modeling of molecular processes, it is essential to determine the context-dependent quantitative protein inventory. Nearly all biological processes, including metabolism, signaling, transport, mechanical functions, and immune responses are mediated by proteins. Hence, precise control of protein expression is fundamental for all organisms during development and to cope with environmental challenges [1, 2]. However, while mRNA concentrations can be readily measured by bulk or single-cell sequencing technologies, protein concentrations often correlate poorly with mRNA concentrations [3, 4]. Sensitivity analyses of quantitative models [5], and evidence from genome-wide association studies, where variants altering gene expression can have major effects on signaling and disease, highlight the importance of understanding protein concentration changes [6]. Thus, accurate determination of protein concentrations in different cell types, in different conditions and of different genetic variants is critical for both mechanistic understanding and effective modeling of biological systems [7].

The experimental measurement of proteomes remains a technically challenging and costly process [8]. By contrast, obtaining systems-level transcriptomic data is both more affordable and common [9]. In the absence of direct protein abundance data, analytical and modeling approaches either rely on experimental determination of protein concentrations for the conditions of interest, which is limited by cost and throughput, or use approximations from transcript concentrations. In silico methods that more accurately model protein concentration are expected to improve the precision of computational analyses. Yet, the complexity of proteostatic regulation currently prevents the scaling of mechanism-based modeling approaches [10–15]. Recent advances in artificial intelligence and machine learning have enabled the development of quantitative predictive models for various challenging biological problems [16–21]. These achievements have been facilitated by the increasing availability of systematic large datasets for training and evaluation [22].

With matched transcriptome-proteome datasets becoming available for both *H. sapiens* [3] and *A. thaliana* [4], we sought to leverage these data to more accurately infer protein concentrations from mRNA concentrations for unseen genes. Earlier machine learning efforts for predicting protein concentrations relied on explicitly defined input features of the mRNA, such as start/stop-codon context, or the protein sequence, such as linear peptide motifs [17, 23]. To reduce assumptions and limit bias, as well as to streamline feature selection, we applied convolutional layers that learn sequence-based features directly. We experimentally optimized the CNN architecture and analyzed its learned weights to identify sequence features most influential for determining PTRs. To begin examining differences and similarities in translational regulation across large evolutionary distances, we developed our models in parallel for both *H. sapiens* and *A. thaliana*.

## Methods

### Datasets

We used expression data from Wang et al. [3] for *H. sapiens* and Mergner et al. [4] for *A. thaliana*. In the *H. sapiens* dataset, transcript abundances were originally normalized as fragments per kilobase million (FPKM), while in the *A. thaliana* dataset they were normalized as transcripts per million (TPM) [24]. Both studies applied a minimum threshold of 1 FPKM or 1 TPM, respectively. To ensure consistency between the datasets we converted the *H. sapiens* transcript data into TPM using the formula TPM = FPKM / Σ(FPKM) * 10^6^ [25]. Proteome measurements in both datasets were given as intensity-based absolute quantification (iBAQ) [26] and were filtered using a minimum intensity threshold of 5,000. Because the *A. thaliana* data were log_2_-transformed, we also applied log_2_-transformations to the *H. sapiens* data.

For sequence data, we used the same database releases employed by the respective studies for RNA-seq and LC–MS/MS experiments, except that the *A. thaliana* untranslated regions (UTRs) were taken from a slightly larger release. Specifically, for *H. sapiens*, we obtained sequence data from Ensembl release 83 [27]. For *A. thaliana*, we used Araport11 release 2016–06 [28] for coding sequence (CDS) and the 2022–09–14 release for UTRs. All sequence data were processed by one-hot encoding and then padded to a uniform length, ensuring that each input to the model had the same dimensionality.

### Machine learning

All experiments were performed in TensorFlow 2.8 [29] using default parameters unless stated otherwise. Each experiment was repeated independently five times, and for each repetition, we applied tenfold cross-validation. We optimized the models with stochastic gradient descent without momentum. Learning rates were tuned based on the type of input features: for codon counts, nucleotide counts, amino acid counts, and start-/stop-codon context, we used a learning rate of 1*10^–3^; for single sequence inputs such as 5' UTR, 3' UTR, CDS, and protein sequence, we used 4*10^–4^; and for experiments that combined multiple features, we used 1*10^–4^. Single-feature experiments were trained for 256 epochs, whereas combined-feature experiments ran for 512 epochs, both with a batch size of 32. We employed a custom NaN-safe mean squared error (MSE) as the loss function to accommodate the varying number of valid data points per gene.

The codon, nucleotide, and amino acid count features were each processed through a log_2_-transformation followed by a dense layer. The start- and stop-codon context features were passed directly to a dense layer. For the 5' UTR, 3' UTR, CDS, and protein sequences, we employed a single convolutional layer containing 16 filters. The filter size was set to 8, 10, or 12 (corresponding to the experiments denoted as 8nt, 10nt, and 12nt, respectively) and was multiplied by the dimensionality of the one-hot encoding, which was 4 for the 5' UTR, 3' UTR, and CDS, and 20 for the protein sequences. Each convolutional layer output then passed through a series of activation and pooling operations, including a tanh activation, ReLU activation, sum-pooling, and a log_2_-transformation, followed by a final dense layer (Fig. 2A, bottom). In the combined-feature experiments, we introduced two intermediate dense layers, the first with 32 filters and the second with 16 filters, before the final dense layer.

In all experiments, the final dense layer consisted of two filters plus a bias term. These two outputs represented the parameters a and b in the equation log_2_(iBAQ) = a * log_2_(TPM) + b (Fig. 2A, top). When additional input genes were included, we expanded the final dense layer to produce (2 + the number of input genes) outputs. This enabled the equation log_2_(iBAQ) = a * log_2_(TPM) + b + δ * log_2_(additionalTPM), where additionalTPM is the TPM vector of the additional input genes. The Python implementation of these models is provided in the Supplementary Source Code.

### Clustering

We performed clustering of the convolutional filters using scikit-learn 1.1 [30] to identify sequence motifs. Each experiment involved standardizing the filters independently. We then applied a cutoff of 0.2 on the standard deviation within each position of the filter to identify positions with sufficient variation in nucleotides or amino acids. After this filtering step, we duplicated, padded, and shifted each filter to generate all possible offset combinations. We clustered the resulting matrices for each feature using OPTICS [31] with a Euclidean distance. For nucleotide input features, we used an xi value of 10^–2^, and for amino acid input features, we used 5*10^–3^. After clustering, we sorted the clusters from largest to smallest and removed redundant filters, ensuring each filter was only represented once despite initial duplications and shifts. For visualization, we scaled the clusters so that their largest peak had a maximum value of 1. We then centered the clusters in this peak and removed nucleotides with values ≤ 0.1.

### Cross-correlation

In the cross-correlation experiment (Fig. 3D), we conducted a tenfold cross-validation with 5 independent repeats. For each repeat and fold, we computed the linear regression and MSE for every possible gene–gene combination. We then averaged these MSE values across all repeats and folds. The resulting output matrices measured 18,200 by 18,200 for *H. sapiens* and 25,285 by 25,285 for *A. thaliana*. We replaced values involving gene pairs with fewer than 21 matched data points with NaN. Subsequently, we specifically searched for pairs with NaN-safe minimum values.

### Gene ontology

We performed functional enrichment analysis using the Panther web service 18.0 [32] and the Gene Ontology database, accessed in May 2023 [33]. We set the annotation to ‘GO biological process complete’, used Fisher’s exact test as the test type, and applied a False Discovery Rate correction.

## Results & discussion

**Fig. 1:**
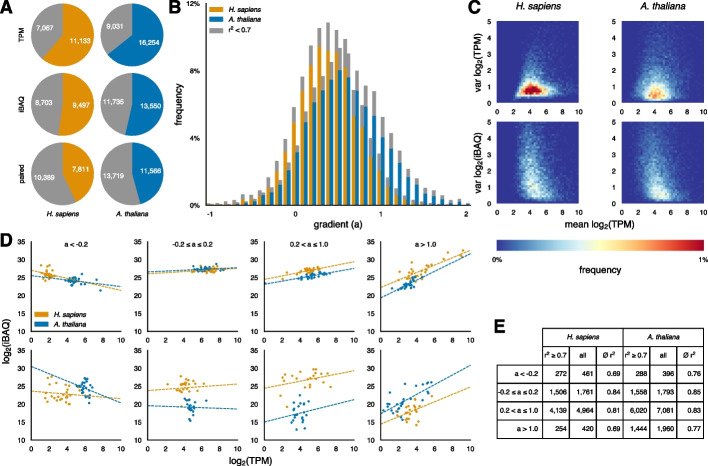
Analysis of matched transcriptome-proteome datasets from *H. sapiens* and *A. thaliana*.** A** Genes grouped based on the number of valid data points: genes with ≥ 20 data points are colored, genes with fewer than 20 are shown in gray.** B** Distribution of linear regression gradients. Poor-quality regressions (r^2^ < 0.7) are shaded in gray. **C** Average transcript abundances plotted against the variance of transcript and protein abundances (left: *H. sapiens*, right: *A. thaliana*). **D** Representative examples within the indicated gradient bin. The top panels show the 100th best-fitting gene, and the bottom panels show the 100th worst-fitting gene. **E** Histogram and average values for genes in the indicated gradient bin

Predicting protein from RNA levels can be accessed at different levels of resolution. We first explored how well the relationship between transcript level and protein level can be predicted from sequence features. We analyzed two matched transcriptome-proteome datasets spanning 29 tissues for *H. sapiens* [3] and 30 tissues for *A. thaliana* [4] (Methods). We began by examining the distribution of data points in both species (Fig. 1A). For each organism, we grouped genes based on their number of matched mRNA-protein data points into two categories: genes with 20 or more data points and those with fewer than 20. To reduce statistical artifacts and enhance robustness, we focused subsequent analyses and training on genes with at least 21 matched data points. This subset comprised 7,606 *H. sapiens* and 11,230 *A. thaliana* genes. Genes represented by exactly 20 matched data points – 205 *H. sapiens* and 336 *A. thaliana* – were reserved as a hold-out set for independent testing.

We first determined which of four commonly used regression methods best captured the relationship between mRNA and protein concentrations. Using five independent repeats of a tenfold cross-validation that excluded tissues, we evaluated prediction quality with the coefficient of determination (r^2^) (Supplementary Fig. 1A). By definition, r^2^ ranges from negative infinity to 1, where 0 corresponds to the mean of the target distribution – in this case, the average protein concentrations across all tissues. Linear regression produced the most reliable predictions and thus served as the baseline for subsequent analyses. More complex regressors, such as quadratic models, were less robust, a likely indication of overfitting due to limited data points.

We also examined the common assumption that changes in transcript abundances directly reflect protein abundance changes. To test this, we calculated a tissue-specific log_2_ iBAQ (iBAQ_SPEC_) value as the product of the average log_2_-transformed protein abundance (iBAQ_AVG_) and the ratio of tissue-specific (TPM_SPEC_) to average (TPM_AVG_) log_2_-transformed transcript abundance (iBAQ_SPEC_ = iBAQ_AVG_ * TPM_SPEC_/TPM_AVG_). This approach yielded negative r^2^ values for both organisms (*H. sapiens*: r^2^ = −1.5; *A. thaliana*: r^2^ = −3.4), suggesting that simply normalizing protein abundances by changes in transcript abundances is inferior to using average protein abundances alone.

To investigate whether linear regression faces inherent limitations, we grouped genes based on their linear regression gradients and classified the resulting fits into two categories: good (r^2^ ≥ 0.7) and poor (r^2^ < 0.7). The distribution of gradients was approximately Gaussian for both species and showed no systematic bias related to fit quality, as both good and poor fits were evenly represented across the range (Fig. 1B). For *H. sapiens*, this distribution was more narrowly centered, which may be due to technical differences in data processing or reflect distinct aspects of translational regulation compared to A. thaliana. Notably, we observed a subset of genes in both species that exhibited negative gradients yet still achieved high r^2^ values.

Exploring how the number of matched data points affects extrapolation quality reveals that too few data points hinder the identification of consistent relationships, but once at least 20 are available, predictions become fairly robust (Supplementary Fig. 1B). Since the total count of matched data points depends on both the number per gene and how many genes populate each bin, excluding genes with fewer matched data points does not notably reduce the available dataset (Supplementary Fig. 1C). Overall, we used 213,444 of 256,551 (83%) available data points for *H. sapiens* and 322,197 of 379,611 (85%) for *A. thaliana* in our training and testing.

We next explored how average transcript abundances influence the variance in both transcript and protein abundances (Fig. 1C). In both *H. sapiens* and *A. thaliana*, the highest variance occurs at log_2_ TPM between 3 and 5. While the average log_2_ TPM variance is significantly lower in *H. sapiens* than in *A. thaliana* (*P* < 2 * 10^–9^, Mann–Whitney U test), the protein variance does not differ substantially (*P* = 0.25, Mann–Whitney U test). Even though low-abundance transcripts were readily detectable, their corresponding proteins were not as easily measured at the lower end of the transcript scale, giving the variance distribution a truncated appearance (Fig. 1D, E).

For illustration, we plotted the 100th best- and worst-fitting gene within the indicated gradient bin for both species. Notably, several genes with a negative gradient (a < −0.2) are not statistical artefacts; instead, they show a clear trend across many data points. Although on average genes with negative gradients have the lowest coefficient of determination, more than half of them still achieve a very robust r^2^ ≥ 0.7. Surprisingly, nearly a quarter of all analyzed genes have gradients close to zero, indicating that increases in mRNA concentration do not substantially affect protein concentration under the measured steady-state conditions. About 85% of these genes display stable protein concentrations, while some poor fits resemble non-linear point clouds, suggesting more complex regulatory mechanisms at play.

Functionally, genes with negative gradient (a < −0.2) and robust fits (r^2^ ≥ 0.7) are associated with core cell regulatory processes in both species [32]. In *H. sapiens*, these genes are enriched in functions such as 'translation’ (FDR = 2.5 * 10^–46^, Fisher’s exact test), ‘gene expression’ (FDR = 6.9 * 10^–31^, Fisher’s exact test), and ‘metabolic process’ (FDR = 8.6 * 10^–24^, Fisher’s exact test). Similarly, in *A. thaliana*, the corresponding functions include ‘protein metabolic process’ (FDR = 4.1 * 10^–7^, Fisher’s exact test), ‘macromolecule metabolic process’ (FDR = 9.6 * 10^–7^, Fisher’s exact test), and ‘intracellular transport’ (FDR = 7.0 * 10^–6^, Fisher’s exact test) (Supplementary Table 1).

To develop a linear regressor for predicting protein abundance – specifically the gradient (a) and offset (b) of the linear relationship between protein to RNA concentrations – we first sought to identify the most informative features. This helped us understand how different parts of the mRNA and the protein sequence contribute to the gradient (representing the PTR). At the same time, it allowed for individual hyperparameter optimization for each input feature.

**Fig. 2:**
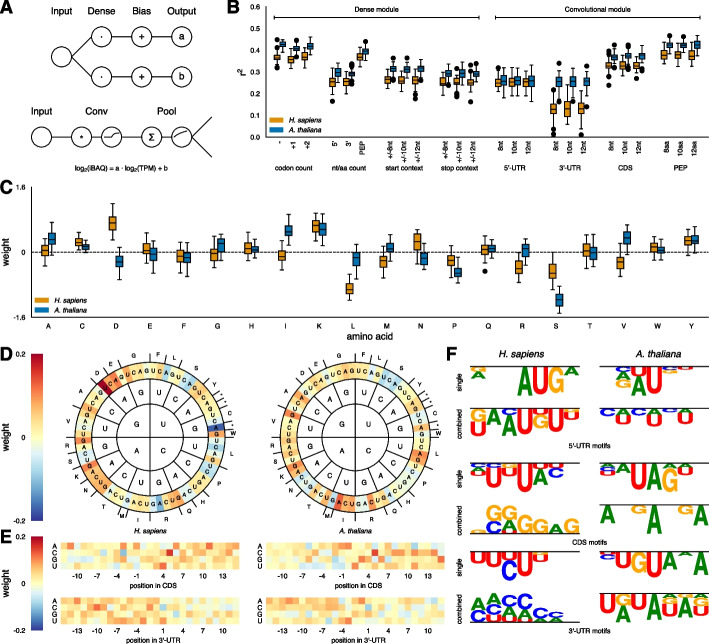
Overview of sequence-based experiments. **A** Schematic representation of the two sequence-based front-end modules. **B** Predictive performance of each sequence input feature during cross-validation. Numbers indicate either the nucleotide shift of the codon count, the range of the context for start-/stop-context, or the filter size of the convolutional layers. **C** Learned weights for amino acid usage in the single-feature model. **D** Learned weights for codon usage in the combined-feature model (left: *H. sapiens*, right: *A. thaliana*). **E** Learned weights for start- and stop-codon context in the combined-feature model (top panels: start-codon context, bottom panels: stop-codon context, left: *H. sapiens*, right: *A. thaliana*). **F** Largest motif clusters identified in both the single-feature model and the combined-feature model for each sequence input feature (left: *H. sapiens*, right: *A. thaliana*)

We implemented two distinct front-end modules, each tailored to different input data types. For histogram-like and fixed-length sequence features (such as codon, nucleotide, and amino acid counts, as well as start-/stop-codon context), we employed two parallel dense layers combined with a bias operator to predict a and b (the ‘dense module’) (Fig. 2A, top). For identifying linear motifs within continuous sequences (5' and 3' UTRs, coding sequences (CDS), and the translated protein sequence (PEP)), we applied a convolutional module that uses sliding windows (Fig. 2A, bottom, Methods), followed by two parallel dense layers combined with bias operators to predict a and b (Fig. 2A, top).

Figure 2B shows the predictive power of each sequence feature during cross-validation. Ultimately, we integrated the modules representing all features into a single model, optionally adding one or two additional dense layers. The combined-feature model with two dense layers achieved an average r^2^ of 0.30 *for H. sapiens* and 0.32 for *A. thaliana* on independent test data. The difference in performance might stem from inconsistencies in data quality, as variations in methodology have been shown to significantly affect mRNA-protein correlations [34]. We also tested whether combining both datasets might leverage any shared patterns in translational regulation. However, training a joint model for both organisms reduced performance in each by more than two percentage points (not shown). Likewise, a minimal model that omitted potentially redundant information showed a lower r^2^, indicating that all sequence features contribute valuable information (not shown).

Among the ‘dense module’ features, codon counts were most informative for *A. thaliana*, while amino acid counts performed best for *H. sapiens*. This finding aligns with previous studies, which identified simple sequence features, such as codon and nucleotide counts, as the most significant predictors of protein concentrations [35]. Somewhat surprisingly, out-of-frame codon counts were only slightly less predictive than in-frame codon counts. Given the strong predictive value of the amino acid counts, it seems unlikely that nucleotide identity alone, independent of coding potential, influences the PTR. A more plausible explanation is that even when the reading frame is shifted, the resulting codon counts still capture underlying trends in overall amino acid composition, albeit in a scrambled form. This interpretation is supported by the observation that a + 2 shift in the reading frame outperforms a + 1 shift. Shifting by + 2 bases preserves the connection between the first two nucleotides of each codon, discarding mainly the less informative wobble position, and thus retains a closer approximation of the amino acid composition.

Among the “convolutional module” features, the coding sequence stands out as the most informative for predicting protein abundance, especially when examined in its translated form. While most features show slightly better performance in *A. thaliana*, the predictive power of 5' UTR motifs is virtually identical for both organisms. In contrast, the 3' UTRs differ substantially in their predictive utility, with human 3' UTRs proving more challenging (Fig. 2B). This may reflect the complexity introduced by the generally longer 3' UTRs in *H. sapiens*, which complicates identifying stable, informative motifs. Notably, the predictive values of the 5' and 3' UTR nucleotide counts from the dense module are indistinguishable, especially in *H. sapiens*.

To pinpoint which features drive protein abundance prediction, we extracted the learned weights for the gradient parameter (*a*) from both the combined and single-feature models across all repeats and folds, visualizing them as averaged heatmaps (Fig. 2C-F, Supplementary Fig. 2A-D). In the combined-feature models, these weights are generally smaller than in the single-feature models. While the overall trends remain similar, some signals become weaker or even disappear in the combined-feature models. This suggests partial redundancy among features, such as between codon and amino acid counts, and that the final dense layer sums all weights without additional scaling. To highlight variations in codon usage and focus on the non-fixed parts of the start- and stop-codon contexts, we truncated the color scales at 1 for the single-feature model visualization.

For most features, we observed both commonalities and clear differences between *H. sapiens* and *A. thaliana*, helping to explain why modeling PTRs jointly for both species reduces predictive performance. Examining amino acid counts, we found that only a few amino acids were strongly informative, even in the single-feature module (Fig. 2C). In *H. sapiens*, the charged aspartate (D) and lysine (K) positively influence PTRs, while leucine (L) and serine (S) have a negative impact. By contrast, in *A. thaliana*, D exerts a negative effect, and isoleucine (I), which shows no effect in humans, has a strong positive impact. Overall, hydrophobic amino acids tend to contribute more positively to PTR predictions in plants than in humans. This may reflect the differing temperature ranges in which these proteins function organisms thrive – 37 °C for humans versus a variable range (4 °C to over 30 °C) for *A. thaliana –* imposing distinct biophysical constraints on protein stability and abundance.

Redundant codons also seem to enable finer tuning of PTRs. For example, both S and arginine (R) are encoded by six codons, and within these sets, certain codons increase PTR while others reduce it. In *H. sapiens*, AAG for R is positively weighted, whereas CGA is negatively weighted. Similarly, in *A.* thaliana, AGC for S is positive, while UCA reduces the PTR (Fig. 2D).

Next, we examined the sequence context of start- and stop-codons in the combined-feature model. In both organisms, the most prominent positive impact arises from a cytosine (C) at the + 5-position relative to the start codon (Fig. 2E, top panels). Intriguingly, a study in yeast that investigated ribosome occupancy and translational efficiency also identified a + 5 C and a weaker enrichment for uracil (U) at the + 4 and + 6 positions [36]. In our data, the effect of the + 5 C is strong in both human and plants, with an additional contribution from U at the + 4 position in humans (Fig. 2E). Furthermore, the presence of adenine (A) before the ATG codon in both species aligns with patterns observed in yeast, where efficiently translated genes also show purines (A or a guanine (G)), in the −3 position, known as the Kozak sequence (A/G)CCAUGG [37]. Our results suggest slight deviations from this canonical motif, with an A at the −1 position and a U at the + 1 position exerting a stronger positive influence than the classic C and G at these positions.

Like the start context, we also see consistent patterns around the stop codon (Fig. 2E, bottom panels). In both organisms, a C at the + 1 position following the stop codon and a G at the + 3 position strongly reduce the predicted PTR. By contrast, substituting a U at the + 1 position increases the PTR in humans.

To further understand the patterns of the convolutional filters, we clustered the learned weights from each repeat and fold, incorporating all possible shifted combinations. After clustering each experiment and species separately, we examined the resulting motifs. Similar to the start- and stop-codon context, the convolutional module recovered numerous known motifs with well-documented biological significance and mechanisms. For example, the CUCUCU motif identified in the 5' UTRs of *A. thaliana* corresponds to a binding site for polypyrimidine tract-binding proteins, which are involved in various aspects of RNA metabolism [38]. In the 3' UTR of *A. thaliana*, the UGUA motif, visible in the single-feature model (Fig. 2F, right, panel 1), is known to bind the cleavage factor I_m_ [39]. Likewise, the UAUAUA motif found in *A. thaliana* 3' UTRs, was previously described in yeast as a stability regulator [40]. In humans, a G-quadruplex motif in the 5' UTR of oncogenes has been shown to modulate translation [41]. Interestingly, the strong weight assigned to this motif in the human CDS suggests that its functional impact may not be strictly confined to the 5' UTR, indicating some positional flexibility in its regulatory role.

Many of the motifs discovered by the convolutional module have been described previously, with their molecular mechanisms already established. Their recurrence here reinforces their importance and suggests that motifs not yet characterized could also be functionally relevant. However, it is crucial to consider that the extracted weights reflect patterns learned by the model rather than direct biological entities. For instance, the highest scoring 3' UTR motif found by the combined-feature model in *A. thaliana* includes both the UAUAUA motif and the UGUA motif identified by the single-feature model. In this way, the extracted weights may represent a blend of functional sequence motifs that influence PTR through diverse regulatory mechanisms.

Overall, we observe substantial variation in the predictive power of different features and only modest improvements when combining all features compared to using the single best feature alone. This finding suggests that we have largely captured the information provided by local one-dimensional sequence features. The remaining unexplained variance likely reflects more complex, context- and environment-dependent regulatory mechanisms, as well as interactions among distant regulatory elements within individual RNA molecules.

**Fig. 3:**
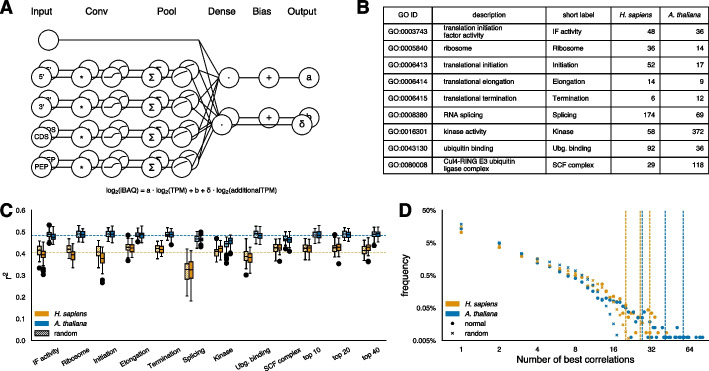
Overview of cell context-specific experiments. **A** Expanded sequence-based model architecture incorporating additional context-dependent input genes. **B** Histogram of manually selected GO terms of potential regulatory genes. **C** Predictive performance of each tested input feature during cross-validation. Dotted lines indicate the best-performing model from previous experiments. **D** Linear cross-correlation of all gene–gene combinations. Dotted lines indicate cutoffs for the top 10, 20, and 40 most correlated genes

We next considered whether additional context-dependent information might improve protein abundance predictions. We modified our model architecture to compute a sequence-based vector δ, which was then used in a dot product with the mRNA concentrations of selected genes (Fig. 3A, Material and Methods). We hypothesized that these genes, potentially involved in proteostasis, or reflective of general cell state, such as kinases, could provide insight into PTR variance (Fig. 3B). As controls, we used randomly selected gene sets of the same size. Surprisingly, none of the tested gene sets improved the model’s prediction in any meaningful way (Fig. 3C). Some sets slightly reduced performance, while the random controls remained unchanged.

Given these results, we asked if it was possible to identify sets of informative input genes analytically, without relying on prior knowledge. We performed linear regression for every possible gene–gene pair, using cross-validation and multiple repeats. For each target gene (output gene), we selected the input gene that best supported protein abundance prediction and recorded how often each input gene appeared as a top correlate across all targets. Plotting the resulting degree distribution (Fig. 3D), we found that while controls produced correlations for up to 18 and 24 genes in *H. sapiens* and *A. thaliana*, respectively, several actual input genes correlated with far more target genes (*P* = 1.1 * 10^–20^ for *H. sapiens* and *P* = 1.2 * 10^–6^ for *A. thaliana*, Mann–Whitney U-test).

We then tested whether the top 10, 20, or 40 most correlated genes as input could improve predictions. Again, this had only minor impact compared to our previous best models (Fig. 3C). Despite the limited gains, we examined the gene ontology (GO) functions of these highly correlated (average r^2^ > 0.8) genes. In *H. sapiens*, they were enriched for immunity-related processes, such as ‘adaptive immune response’ (FDR = 2.1 * 10^–5^, Fisher’s exact test), ‘regulation of immune system process’ (FDR = 1.9 * 10^–4^, Fisher’s exact test) and ‘immune effector process’ (FDR = 2.3 * 10^–3^, Fisher’s exact test). By contrast, in *A. thaliana,* top functions included ‘response to stimulus’ (FDR = 7.2 * 10^–7^, Fisher’s exact test) and specifically ‘response to light stimulus’ (FDR = 2.0 * 10^–4^, Fisher’s exact test), suggesting that environmental factors strongly influence protein abundances (Supplementary Table 2). These results hint that the predominant challenges faced by each organism – environmental variability for plants and immune challenges for mammals – may shape the underlying regulatory landscape of protein homeostasis.

We suspect that insufficient data is limiting our ability to predict protein abundances from transcript abundances. Although we have thousands of matched transcript-protein pairs for identifying sequence features, the number of tissues is restricted to 29 for *H. sapiens* and 30 for *A. thaliana*. Even if traditional guidelines like the “rule of ten” (ten times more data points than variables) are not strictly applicable, a model built from only 29 conditions can effectively utilize at most 29 variables. In contrast, a proteins interaction network context involves roughly 20,000 other genes, and capturing condition-specific interaction network effects on protein abundance would therefore require thousands of condition-specific matched datasets – three orders of magnitude more than currently available.

## Conclusions

We developed a CNN-based model to predict protein abundance and identify relevant sequence features, achieving r^2^ = 0.30 for *H. sapiens* and r^2^ = 0.32 for *A. thaliana*. It is important to note that our coefficient of determination was calculated independently for each gene, rather than across tissues, avoiding some of the misleading interpretations seen in previous work [42]. Despite the limitations of interaction network-based approaches, our model surpasses earlier sequence-based efforts by providing a framework that can be extended to account for alternative, previously unseen sequences, enabling more precise mRNA-dependent protein abundance predictions.

For *H. sapiens*, our model improves prediction performance by nearly 50% compared to previous sequence-based methods (r^2^ = 0.19 [43] and r^2^ = 0.22 [23]). Moreover, to our knowledge, our model is the first sequence-based predictor for *A. thaliana*, establishing a benchmark for plant studies. In *Saccharomyces cerevisiae*, previous attempts to predict protein abundances independent of transcript abundances have reached r^2^ = 0.48 [44]. For *H. sapiens*, interaction network-based methods applied to cancer cells averaged Pearson correlation coefficients of r = 0.49 (r^2^ = 0.24) [45], and a tailored ovarian cancer model reached r = 0.60 (r^2^ = 0.36) [46]. Notably, this study reported that incorporating the mRNA abundances of all protein interaction partners improved predictions – a factor we did not consider here.

Our combined sequence and abundance-based approach may be approaching the limit imposed by the current training data and might be susceptible to overfitting. Future work should explore integrating CNNs and other deep learning models with more extensive training data, as well as considering complex regulatory interactions beyond those examined here. Moreover, for the cross-validation genes were randomly distributed among training- and validation sets, and it is possible that homologous sequences cause data leakage resulting in slightly increased performance measures. The reidentification of experimentally validated sequence motifs suggest that not only correlated, but indeed causal sequence motifs were identified. Nonetheless, future expansion of this work will aim to eliminate this issue, e.g. as proposed [47]. At the same time, it is telling that various approaches plateau at similar performance levels, suggesting that current unknown factors and constraints will require new conceptual frameworks or additional data to achieve further improvements in predictive power.

The fact that our sequence-based model performs equally well on the more challenging, RNA-dependent PTR in multicellular *A. thaliana* underscores its robustness. It also supports the notion that approximately one-third of the variation in protein concentrations is determined by intrinsic sequence features, while the remaining two-thirds arise from higher-level regulatory mechanisms. To model these more complex factors effectively, additional training data or a better understanding of the underlying regulatory principles will be needed.

By examining the trained neural network, we identified key input features that not only improve prediction accuracy but also mirror known molecular regulatory mechanisms, such as those governing translation initiation or mRNA stability. To address the data limits in condition-specific predictions, it may be feasible to constrain gene sets based on protein–protein [5, 48, 49] or protein-RNA [50, 51] interaction networks. Such an approach could capture a reasonable subset of the regulatory landscape, thereby reducing the combinatorial complexity and improving predictive performance.

## Supplementary Information

Supplementary Material 1: Supplementary Fig. 1. Extended regression analysis. A Prediction scores of different regression methods. B Prediction scores of the linear regressor as a function of the number of matched data points. C Frequency distributions of genes and their matched data points.

Supplementary Material 2: Supplementary Fig. 2. Extended overview of sequence-based experiments. A Learned weights for amino acid usage in the combined-feature model. B Learned weights for codon usage in the single-feature model (left: H. sapiens, right: A. thaliana). C Learned weights for start- and stop-codon context in the single-feature model (top panels: start-codon context, bottom panels: stop-codon context, left: H. sapiens, right: A. thaliana). D Second largest motif clusters identified in both the single-feature model and the combined-feature model for each sequence input feature (left: H. sapiens, right: A. thaliana).

Supplementary Material 3: Supplementary Table 1. Functional analysis of genes with a negative gradient (a < -0.2) and robust fits (r2 ≥ 0.7). A Raw linear regressions values for H. sapiens. B Raw linear regressions values for A. thaliana. C Gene Ontology enrichment analysis for H. sapiens. D Gene Ontology enrichment analysis for A. thaliana.

Supplementary Material 4: Supplementary Table 2. Functional analysis of genes with good average fits (r2 ≥ 0.8). A Raw linear regressions values for H. sapiens. B Raw linear regressions values for A. thaliana. C Gene Ontology enrichment analysis for H. sapiens. D Gene Ontology enrichment analysis for A. thaliana.

Supplementary Material 5: Supplementary Table 3. Calculated linear parameters and predictions for: A H. sapiens. B A. thaliana.

Supplementary Material 6: Source Code.

## Data Availability

No datasets were generated or analysed during the current study.

## Abbreviations

A: Adenine
*A. thaliana*: *Arabidopsis thaliana*
C: Cytosine
CDS: Coding sequence
CNN: Convolutional neural network
D: Aspartate
FDR: False discovery rate
FPKM: Fragments per kilobase million
G: Guanine
GO: Gene ontology
*H. sapiens*: *Homo sapiens*
I: Isoleucine
iBAQ: Intensity based absolute quantification
K: Lysine
L: Leucine
NaN: Not-a-number
PEP: Protein sequence
PTR: Protein-to-mRNA ratio
R: Arginine
r: Pearson correlation coefficient
r^2^: Coefficient of determination
S: Serine
TPM: Transcripts per million
U: Uracil
UTR: Untranslated region
