# Supplementary figures and images for: Inferring protein from transcript abundances using convolutional neural networks
Source: BioData Min. 2025 Feb 27;18:18. doi: 10.1186/s13040-025-00434-z (PMC11866710; doi:10.1186/s13040-025-00434-z)

**A**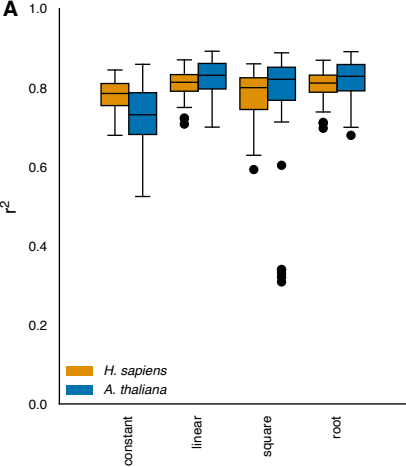**B**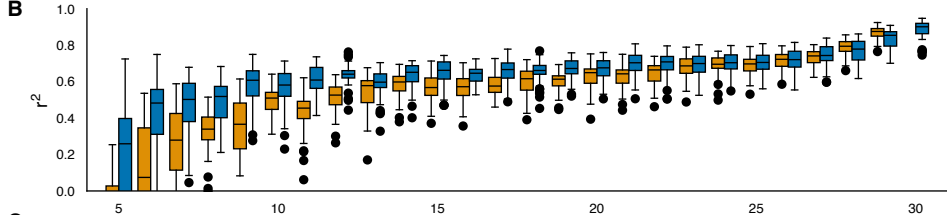**C**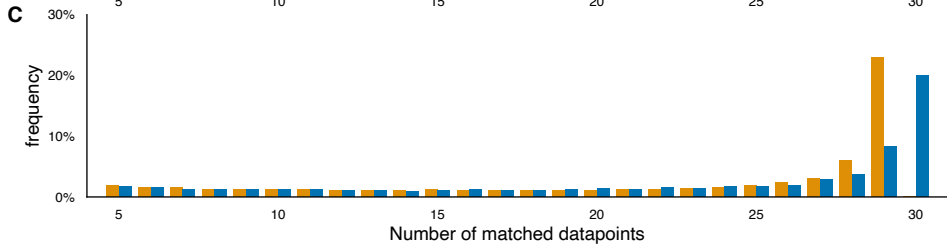

Supplement: Supplementary file 1 — Supplementary Material 1: Supplementary Fig. 1. Extended regression analysis. A Prediction scores of different regression methods. B Prediction scores of the linear regressor as a function of the number of matched data points. C Frequency distributions of genes and their matched data points. [file 13040_2025_434_MOESM1_ESM.pdf]

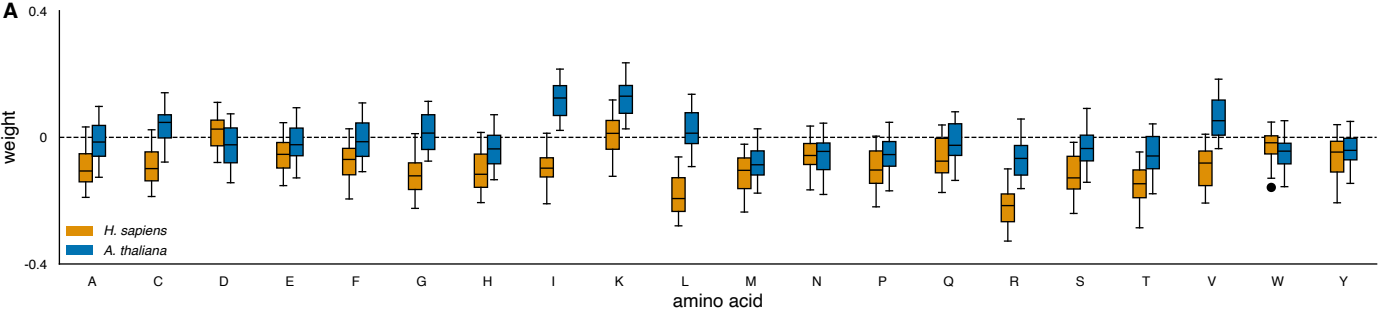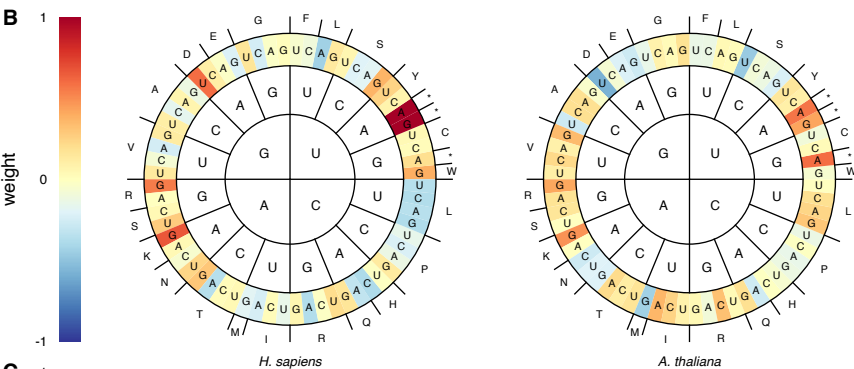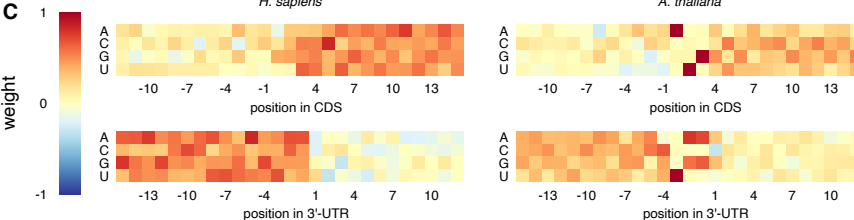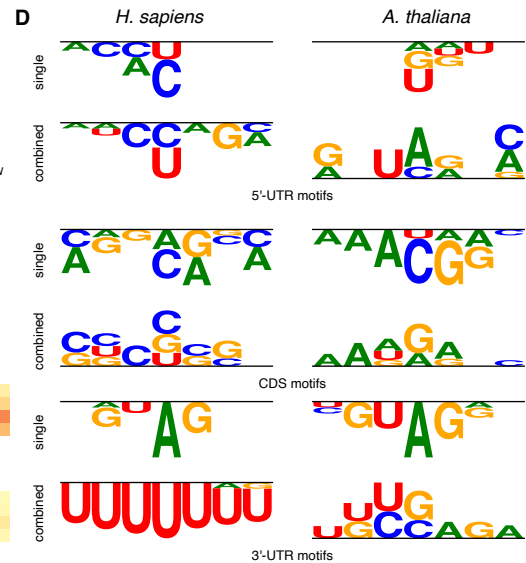

Supplement: Supplementary file 2 — Supplementary Material 2: Supplementary Fig. 2. Extended overview of sequence-based experiments. A Learned weights for amino acid usage in the combined-feature model. B Learned weights for codon usage in the single-feature model (left: H. sapiens, right: A. thaliana). C Learned weights for start- and stop-codon context in the single-feature model (top panels: start-codon context, bottom panels: stop-codon context, left: H. sapiens, right: A. thaliana). D Second largest motif clusters identified in both the single-feature model and the combined-feature model for each sequence input feature (left: H. sapiens, right: A. thaliana). [file 13040_2025_434_MOESM2_ESM.pdf]
